# Mean Bone Density in Interadicular Areas of Maxilla among Patients Visiting Dental Unit of a Tertiary Care Centre: A Descriptive Cross-sectional Study

**DOI:** 10.31729/jnma.8037

**Published:** 2023-02-28

**Authors:** Hemant Kumar Halwai, Ranjit Haridas Kamble, Sumit Kumar Yadav, Sandeep Kumar Gupta, Raju Shrestha, Pallavi Daigavane, Rizwan Gilani

**Affiliations:** 1 Department of Orthodontics and Dentofacial Orthopedics, Universal College of Medical Sciences and Teaching Hospital - College of Dental Surgery, Siddhartha Nagar, Bhairahawa, Nepal; 2 Department of Orthodontics and Dentofacial Orthopedics, Sharad Pawar Dental College and Hospital, Sawangi, Maharashtra, India

**Keywords:** *hone density*, *prevalence*, *prostheses and implants*

## Abstract

**Introduction:**

A close relationship occurs between the type of bone density and the success of orthodontic mini-implant. The aim of this study was to find out the mean bone density in interradicular areas of the maxilla among patients visiting dental unit of a tertiary care centre.

**Methods:**

A descriptive cross-sectional study was performed at the Department of Orthodontics and Dentofacial Orthopedics at a tertiary care centre from 15 January 2022 to 28 June 2022 after taking ethical approval from the Institutional Review Committee (Reference number: UCMS/IRC/175/21). Data was collected from scan reports obtained with a computed tomography scanner. Bone density was measured at 6 mm height from the alveolar crest. Convenience sampling was done. Point estimate and 95% Confidence Interval were calculated.

**Results:**

Out of 70 patients, mean bone density at interradicular areas of maxilla was 992.31±204.20 HU (944.46-1040.13, 95% Confidence Interval). Between central and lateral incisor 50 (71.44%) had D2 type of bone density.

**Conclusions:**

The mean bone density in inter radicular areas of the maxilla among patients visiting the dental outpatient department was similar to other studies done in similar settings.

## INTRODUCTION

In orthodontic practice, anchorage control has typically been a difficult and unpredictable challenge. To overcome this challenge, there have been numerous attempts to obtain bone tissue support for successful anchorage control.^1^ An implant is a mechanical device made from one or more biomaterials that are intentionally placed within the body either totally or partially buried beneath an epithelial surface.^2^ The use of implants for orthodontic purposes started in 1945.^3^

A major limitation of orthodontic mini-implants is loosening and subsequent failure which could lead to premature removal and longer treatment time.^4^ Three main factors that affect the success of dental implants are host, implant, and surgical methods.^5^ A close relationship is shown between types of bone density and the success of dental implants.^6^

The objective of this study was to find out the mean bone density in interradicular areas of the maxilla among patients visiting the dental unit of a tertiary care centre.

## METHODS

This descriptive cross-sectional study was conducted from 15 Jan 2022 to 28 Jun 2022 in the Department of Orthodontics and Dentofacial Orthopedics at the Universal College of Medical Sciences (UCMS). Ethical approval was taken from the Institutional Review Committee (Reference number: UCMS/IRC/175/21) of UCMS. Patients who visited UCMS for Computed tomography (CT) scans for diagnostic purposes after obtaining informed consent were included in the study. Either male or female with ages ranging from 15-40 years of age, no history of previous orthodontic treatment, all permanent teeth present except 3^rd^ molar, and good quality of CT images. The exclusion criteria were: history of any metabolic disorder affecting bone density, pathologic lesions in the jaw, patients with signs of periodontal disease, and alveolar bone loss, history of medication that affects bone density, patients with prosthetic crowns and restorations, and trauma with fracture line passing through the site of bone density measurement. Convenience sampling method was used. The sample size was calculated using the following formula:


$$\begin{array}{ll} \text{n} & ={\text{Z}}^{2}\times \frac{\sigma^{2}}{{\text{e}}^{\text{2}}}\\ & =1.96^{2}\times \frac{83.33}{20^{2}}\\ & =67 \end{array}$$

Where,

n = minimum required sample size
Z = 1.96 at 95% Confidence Interval (CI)
σ = standard deviation calculated from range (minimum and maximum values 1020 and 1520 taken from published literature)^7^
e = margin of error

Minimum required sample size of 67 was calculated. However, we have included 70 patients for the study.

All the scans were obtained with a CT scanner (Brivo CT385, GE Healthcare). The software for 3-Dimesional (3D) visualization of the CT images, multiplanar reconstruction, and bone density measurement used was Osirix, version 5.8.2. Scans were made according to the following technical protocol: 16-slice spiral CT scanner, 120 kV, 120 mAs, 0.6 mm thick slice, ultrahighresolution, and 0° gantry angulation. The scanner was calibrated daily before it was used for the first patient, according to the manufacturer's guidelines. After scanning, Digital Imaging and Communication in Medicine (DICOM) images were created. The acquired 2-dimensional CT DICOM data were then input into a personal computer (MacbookPro,macOS Sierra, version 10.12.5). A 3-D multiplanar reconstruction was performed using the software (Osirix, version 5.8.2). In addition to the axial tomograms, reconstruction was made in coronal, sagittal and oblique planes to easily locate the required site of the inter radicular bone. The bone density was measured in Hounsfield Unit (HU) in the inter radicular area between the right second molar to the left central incisor at a height of 6 mm from the crest of the alveolar bone in the buccal cortical plate of the maxilla using a linear measurement tool. The density value of the region of interest (ROI) was taken for each site and classified under various types of Bone density according to Misch classification into D1 (>1250 HU), D2 (850-1250HU), D3 (350-850 HU) and D4 (15-350 HU).^6^

Data were analysed using IBM SPSS Statistics version 17.0. Point estimate and 95% CI were calculated.

## RESULTS

Among 70 patients, mean bone density at interradicular areas of maxilla was 992.31±204.20 HU (944.461040.13, 95% CI). The lowest mean bone density was found between the first and second premolar with the mean density of 893.7±203 while the highest bone density was found between central incisors with the mean density of 1109.7±274.2 (Table 1).

**Table 1:** Mean bone density at the height of 6 mm in different interradicular areas of maxilla (n= 70).

Bone Density type	Mean±SD
Between central incisor	1109.78±274.10
Between lateral and central incisor	1024.00±145.32
Between lateral and canine	1048.71±221.75
Between canine and 1^st^ premolar	997.00±18410
Between 1^st^ and 2^nd^ premolar	893.70±203.00
Between 2^nd^ premolar and 1^st^ molar	951.10±158.28
Between 1^st^ molar and 2^nd^ molar	921.91±242.91

Between central and lateral incisor, 50 (71.44%) had D2type of bone density, between lateral and canine 12 (17.15%) had D1 type of bone density (Table 2).

**Table 2:** Types of bone density at the height of 6 mm in different inter radicular areas of maxilla (n= 70).

Bone Density type	D1 n (%)	D2 n (%)	D3 n (%)	D4 n (%)
Between central incisor	9 (12.86)	46 (65.71)	15 (21.43)	-
Between lateral and central incisor	10 (14.28)	50 (71.44)	10 (14.28)	-
Between lateral and canine	12 (17.15)	44 (62.85)	13 (18.58)	1 (1.42)
Between canine and 1^st^ premolar	8 (11.42)	42 (60)	20 (28.58)	-
Between 1^st^ and 2^nd^ Premolar	3 (4.28)	40 (57.14)	27 (38.58)	-
Between 2^nd^ premolar and 1^st^ molar	5 (7.14)	40 (57.14)	25 (35.72)	-
Between 1^st^ molar and 2^nd^ molar	5 (7.14)	33 (47.14)	32 (45.72)	-

## DISCUSSION

In the maxillary buccal area, the mean cortical bone density ranged from mean bone density at interradicular areas of maxilla was 992.3±204.2 HU at 6 mm in height from the alveolar crest. Many previous studies^4,7-9^ report a similar range of bone density in the maxilla compared to this study. But some of the studies have found considerably less bone density values than this study.^10-13^ The studies that reported the lower density were performed on dental implants in older age group patients with the edentulous jaw. The present study was conducted to study the prevalence of D2 cortical bone at different buccal inter radicular sites of the maxilla. However, this study consisted of samples with no missing teeth except the third molar and the patients were of a younger age group which could be a factor in the variation of bone density.

In the present study, mean bone density at different heights of the maxillary buccal cortex was found to be in the order: of 6 mm >8 mm. At most of the inter radicular sites of the maxillary buccal cortex, a decreasing trend of bone density was noticed from the coronal to the apical area. It also found that there was a significant decrease in bone density according to height.^8^ Another research reported that in the anterior region most of the sites at the alveolar crest level exhibited significantly higher bone density than the apical level,^4^ but the opposite scenarios were seen in the posterior region.^14^ Similarly, reported that the density increased significantly farther from the alveolar crest.^4^

Bone density is an important parameter in treatment planning for implant placement.^6,15^ Most of the failure of orthodontic mini-implant occurs during the initial stage. Hence, it is important to ensure their primary stability.^16^ During the initial stage, bone density is the key determinant for the primary retention of mini-implants as it is achieved by mechanical means rather than osseointegration.^14^ During the loading of the orthodontic force on mini-implants, stress is distributed on the areas where the bone is in contact with the implant. Bone density influences the area of contact between the implant and bone surfaces. There is greater stress if the area of contact between the two is smaller. Regions of D1 to D3 bones are adequate for mini-implant insertion. Mini-implants placed in D1 and D2 bone exhibit lower stress at the screw bone interface and may provide greater stationary anchorage during loading. Placement in D4 bone is not recommended owing to the high failure rate associated with it.^17^ In the present study type of bone density in the majority of inter radicular sites of the maxilla at different heights was found to be D2 followed by D3 and D1 type of bone respectively. The study showed that the maxilla consisted of D2 and D3 bone whereas the mandible consisted of D1 and D2 bone.^14^ According to the study,^18^ D1 is primarily found in the anterior mandible buccal shelf and mid-palatal region, D2 is primarily found in the anterior maxilla, mid-palatal region, and posterior mandible, and D3 is found primarily in the posterior maxilla and mandible. D4 is found primarily in the tuberosity region. A finding of the present study was similar to this study.

The limitation of this study was that it did not take into consideration the change in bone density among different vertical skeletal patterns. Similarly, other variables like age, sex, and ethnicity were not considered in this study. As this was a single-centric study, the findings of the study cannot be generalized to the large population.

## CONCLUSIONS

Mean bone density in interradicular areas of the maxilla among patients visiting the dental outpatient department was similar to other studies done in similar settings. D2 type of bone density was found in the majority of subjects in all areas of Maxilla. There was a decreasing tendency of mean bone density from coronal to the apical area in most of the inter radicular sites of maxillary buccal cortical bone.

## References

[1] Lim HJ, Eun CS, Cho JH, Lee KH, Hwang HS (2009). Factors associated with initial stability of miniscrews for orthodontic treatment.. Am J Ortho d Dentofacial Orthop..

[2] Williams DF (1987). Definition of Biomaterials: Proceedings of a Consensus Conference of the European Society for Biomaterials.. Elsvier.

[3] Gainsforth BL, Higley LB (1945). A study of orthodontic anchorage possibilities in basal bone.. Am J Orthod Oral Surg..

[4] Ohiomoba H, Sonis A, Yansane A, Friedland B (2017). Quantitative evaluation of maxillary alveolar cortical bone thickness and density using computed tomography imaging.. Am J Orthod Dentofacial Orthop..

[5] el Askary AS, Meffert RM, Griffin T (1999). Why do dental implants fail?. Part I. Implant Dent..

[6] Turkyilmaz I, Tumer C, Ozbek EN, Tözüm TF (2007). Relations between the bone density values from computerized tomography, and implant stability parameters: a clinical study of 230 regular platform implants.. J Clin Periodontol..

[7] Chugh T, Ganeshkar SV, Revankar AV, Jain AK (2013). Quantitative assessment of interradicular bone density in the maxilla and mandible: implications in clinical orthodontics.. Prog Orthod..

[8] Choi JH, Park CH, Yi SW, Lim HJ, Hwang HS (2009). Bone density measurement in interdental areas with simulated placement of orthodontic miniscrew implants.. Am J Orthod Dentofacial Orthop..

[9] Samrit V, Kharbanda OP, Duggal R, Seith A, Malhotra V (2012). Bone density and miniscrew stability in orthodontic patients.. Aust Orthod J..

[10] Shapurian T, Damoulis PD, Reiser GM, Griffin TJ, Rand WM (2006). Quantitative evaluation of bone density using the Hounsfield index.. Int J Oral Maxillofac Implants..

[11] Turkyilmaz I, Tozum TF, Tumer C (2007). Bone density assessments of oral implant sites using computerized tomography.. J Oral Rehabil..

[12] Fuh LJ, Huang HL, Chen CS, Fu KL, Shen YW, Tu MG, Shen WC, Hsu JT (2010). Variations in bone density at dental implant sites in different regions of the jawbone.. J Oral Rehabil..

[13] Hiasa K, Abe Y, Okazaki Y, Nogami K, Mizumachi W, Akagawa Y (2011). Preoperative computed tomography-derived bone densities in hounsfield units at implant sites acquired primary stability.. ISRN Dent..

[14] Chun YS, Lim WH (2009). Bone density at interradicular sites: implications for orthodontic mini-implant placement.. Orthod Craniofac Res..

[15] Turkyilmaz I, Ozan O, Yilmaz B, Ersoy AE (2008). Determination of bone quality of 372 implant recipient sites using Hounsfield unit from computerized tomography: a clinical study.. Clin Implant Dent Relat Res..

[16] Cha JY, Kil JK, Yoon TM, Hwang CJ (2010). Miniscrew stability evaluated with computerized tomography scanning.. Am J Orthod Dentofacial Orthop..

[17] Kravitz ND, Kusnoto B, Tsay TP, Hohlt WF (2007). The use of temporary anchorage devices for molar intrusion.. J Am Dent Assoc..

[18] Misch CE, Kircos LT (1999). Contemporary Imlant Dentistry..

